# Pulmonary Alveolar Microlithiasis: A Case Report with Emphasis on Imaging Findings

**DOI:** 10.1155/2010/819242

**Published:** 2010-07-04

**Authors:** Guilherme Abdalla, Edson Marchiori, Gláucia Zanetti, Antonio Mucillo, Mariana Leite Pereira, Nina Ventura, Pedro Martins, Carolina Pesce Lamas Constantino, Rodrigo Canellas, Viviane Brandão, Romulo Varella de Oliveira

**Affiliations:** ^1^Department of Radiology, Rio de Janeiro Federal University, CEP 21941.913, Rio de Janeiro, Brazil; ^2^Department of Radiology, Fluminense Federal University, CEP 24220.071, Rio de Janeiro, Brazil

## Abstract

Pulmonary alveolar microlithiasis (PAM) is a rare disease characterized by the presence of small calculi in the alveolar space. The authors report a case of a 21-year-old man with a 2-year history of shortness of breath on exertion and dry cough. Physical examination was altered only for crackles at auscultation. Pulmonary function revealed a mild restrictive ventilatory defect and the chest radiograph demonstrated paracardiac confluence of dense micronodular infiltrate. High-resolution CT scan revealed diffuse ground glass attenuation and septal thickening, more pronounced in lower pulmonary regions, with calcifications along the interlobar septa and subpleural regions. A transbronchial lung biopsy confirmed the diagnosis of PAM.

## 1. Introduction

Pulmonary alveolar microlithiasis (PAM) is an uncommon chronic disease characterized by multiple microscopic calculi within the alveoli and a paucity of symptoms in contrast to the imaging findings [1–7]. Symptoms start at most cases in the third and fourth decades of life. It is mostly accepted now that the disease has an autosomal recessive inheritance [2, 5, 8]. Recently, a few reports have described the role of mutation in the type IIb sodium-phosphate cotransporter gene (SCL34A2 gene) in the disease pathogenesis [9, 10]. The authors present a case of symptomatic PAM in a 21-year-old man, emphasizing the role of high-resolution lung CT (HRCT) in the diagnosis of this unusual disease.

## 2. Case Report

A 21-year-old male presented with a 2-year history of progressive shortness of breath on exertion and dry cough. At physical examination, auscultation of the lungs has revealed random wheezes and coarse crackles. Cardiac auscultation was normal, and no cyanosis or peripheral edema was observed. There was no history of smoking or previous known pulmonary disease. On routine blood examination, blood counts and serum chemistries were found to be normal. Arterial blood gas analysis and echocardiography showed no important abnormalities. 

**Figure 1:**
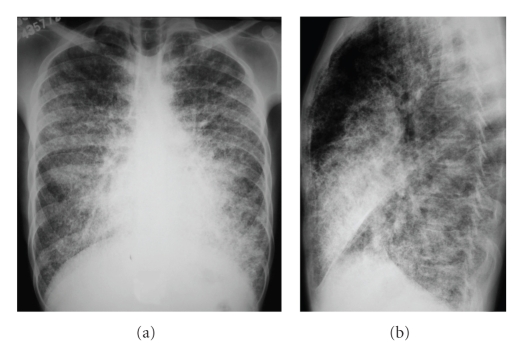
Chest radiographs in anteroposterior (a) and lateral (b) incidences shows a diffuse symmetric lung lesion with confluence of dense micronodular infiltrate. Note the, predominance of the lesions in the paracardiac regions of the lungs.

**Figure 2:**
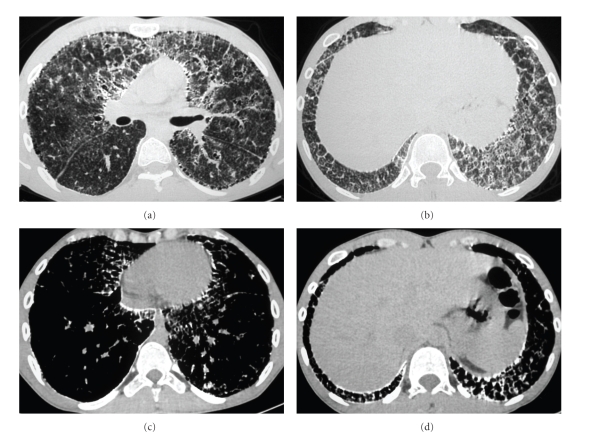
(a-b) High-resolution CT scan reveals diffuse ground glass attenuation and septal thickening, more pronounced in lower pulmonary regions. Note also subpleural cysts. (c-d) Mediastinal window scans shows calcifications along the interlobar septa and subpleural regions.

 Pulmonary function tests (PFT) showed a mild restrictive ventilatory defect, with a reduced total lung capacity of 79% (5.94 L), forced vital capacity of 80% (4.18 L) and a forced expiratory volume in one second of 83% (3.72 L). The sputum was negative for acid-alcohol resistant bacillus and human immunodeficiency virus testing was negative as well. The chest plain films revealed a diffuse symmetric dense bilateral micronodular pattern (Figure 1). Based on this finding, HRCT scan was obtained, revealing diffuse ground glass attenuation and septal thickening, more pronounced in lower pulmonary regions, with calcifications along the interlobar septa and subpleural regions. Subpleural cysts were also noticed (Figure 2). The patient underwent a fiberoptic bronchoscopy with bronchoalveolar lavage and transbronchial lung biopsy. The lavage fluid was negative for tuberculosis or fungi. Microliths were not found. Histology revealed round, concentrically laminated microliths in the alveoli associated with slightly thickened interstitial septa, consistent with the diagnosis of PAM.

## 3. Discussion

PAM is a rare disease that presents chronic evolution, poorly defined etiology and pathogenesis, and is basically characterized by numerous small calculi (denominated calciferites, calcospherites or microlites) within air spaces [1–7, 11, 12]. Patients may remain asymptomatic for many years and do usually become symptomatic between the third and fourth decades [3, 6, 12]. At clinical presentation patients usually demonstrate a lung disorder with restrictive pattern [3, 5–7, 12], as seen in our case. Adult patients commonly show progressive deterioration of the pulmonary function and death usually occurs in mid-life because of respiratory failure associated with *cor pulmonale *[7, 13, 14]. Mean age at the time of diagnosis in the literature is 35 years and apparently there is no important predominance of gender [2, 4, 12], The disease presents a high incidence of familial occurrence (approximately one-third of the cases) suggesting an autosomal recessive inheritance pattern [2, 5, 8, 12]. Chest X-ray of the parents and brother of the patient in our case showed no signs of PAM. Recently, a few reports have described the role of mutation in the type IIb sodium-phosphate cotransporter gene (SCL34A2 gene), which is involved in phosphate homeostasis in several organs, including the lung [9, 10]. SCLC34A2 expression is observed in type II alveolar cells. These cells use phospholipids to produce surfactant and are also responsible for recycling and degrading the outdated surfactant. It is believed that the dysfunction in SCLC34A2 may reduce the clearance of the phosphate released in this process, leading to the formation of microliths. For this reason, it is now accepted that this mutation is responsible for the pathologic changes seen in the pulmonary parenchyma in PAM [10]. Disodium etidronate, which inhibits microcrystal growth of hydroxyapatite and thus inhibits ectopic calcification, has been used to treat PAM. Although some reports show little or no benefit with the use of disodium etidronate [11, 12, 14, 15], a recent study, regarding the long-term results of the treatment in two cases, demonstrated an improvement at the patients PFT and radiological changes [16]. Besides that, some authors demonstrated the usage of measuring the serum concentration of surfactant protein-A (SP-A) and surfactant protein-D (SP-D) in patients with PAM. Alveolar type II cells and Clara cells produce these two proteins in the lungs. The diffuse parenchyma fibrosis, consequent to PAM, causes an increase in permeability, leading to an increase in the levels of these two proteins in the blood. Therefore, SP-A and SP-D measurement may be an alternative to monitor the progression and activity of the disease. [17]. These exams were not performed in our patient. Nevertheless, no effective treatment for end stage PAM currently exists, with the exception of lung transplantation. Until then, seven successful cases have been reported, with no recurrence of the disease. Transplantation should be considered in cases where either severe respiratory failure or right heart failure are present. Patients who undergone lung transplantation have showed an increase in right ventricular ejection fraction and regression of right ventricular hypertrophy [14]. 

 In the radiological diagnosis of PAM, chest radiographs usually reveal diffuse, bilateral areas of micronodular calcifications (‘‘sand storm”) that predominate in the middle and lower lung areas [2–7]. The heart borders and the diaphragm are usually obliterated. Other typical finding includes a black pleural line, demonstrated as an area of increased translucence between the lung parenchyma and the ribs [2, 4, 5, 7]. The chest radiographs of our patient showed a diffuse symmetric lung lesion with dense micronodular aspect, corroborating the pattern described in the literature. 

 The HRCT findings in patients with alveolar microlithiasis vary considerably [2–7]. Ground-glass opacities are the most common finding described in literature [2, 4, 5, 7]. This pattern occurs probably due to small calculi in the air space. Subpleural linear calcification, confluent and diffuse calcified nodules and dense consolidations are also a common finding. Calcifications along the bronchovascular bundles and at the central region of the bronchovascular tree can also be seen [2–7]. The CT scan confirms a predominance of symmetric calcifications along the heart borders and in the lower posterior portions of the lung [2–4, 7]. Small thin-walled subpleural cysts are described as well, and they are responsible for the “black pleura” sign seen in the chest X-rays [2, 4, 5, 11]. Recently, it was described the unique characteristics of the mosaic pattern seen in alveolar microlithiasis, in which the interlobular septa are of calcium density due to the deposition of calcipheriths within the peripheral lobular parenchyma, adjacent to the septa. Some authors consider this pattern very specific and even pathognomonic of PAM on the HRCT scan, since this aspect has not been described in any other disease [2, 7]. Clinicians should have in mind that some findings seen in PAM, such as nodular calcifications, can be found in other diseases like tuberculosis, metastatic osteosarcoma, amyloidosis and silicoproteinosis. Besides that, dense consolidations can also be found in metastatic pulmonary calcification, talcosis and amiodarone lung toxicity. In this way, associated CT findings and clinical features should always be correlated, since these diseases have different kinds of presentation and evolution [18]. 

 In conclusion, PAM is a rare disease that can affect young patients, with chronic and deteriorating evolution. Clinicians should be aware of it existence and the radiological features associated. The micronodular pattern seen in chest radiography can sometimes be misdiagnosed as miliary tuberculosis or other diseases that present with this pattern. In this way, HRCT should always be performed since it can reveal characteristic patterns of alveolar microlithiasis, reserving lung biopsy for atypical and inconclusive cases.
